# Supplementary material for: Orbital textures and evolution of correlated insulating state in monolayer 1T phase transition metal dichalcogenides
Source: Nat Commun. 2025 Apr 22;16:3784. doi: 10.1038/s41467-025-59228-w (PMC12015590; doi:10.1038/s41467-025-59228-w)
Supplement: Supplementary file 1 — Supplementary information [file 41467_2025_59228_MOESM1_ESM.pdf]

Orbital textures and evolution of correlated insulating state in monolayer 1T phase transition metal dichalcogenides

Supplementary Note 1. Surface doping and quantum spin liquid behavior

Mott insulator will exhibit quantum spin liquid behavior when the quantum fluctuations are strong enough to suppress the spin ordering[1,2,3]. Such a state can be revealed by probing the response of magnetic impurities doping[4,5]. We have employed in situ surface doping of Co on the monolayer films (Supplementary Fig. 5). Increasing amounts of Co doping causes the reduced intensity of the LHB and the closing of the CDW gaps in monolayer $TaSe_2$ at 10 K. This doping effect is more like a charge redistribution around the LHB and cannot be described by a rigid shift of the bands, as the Se bands shift further away from the Fermi level. The situation is more complicated for monolayer $NbSe_2$ as the gap is enlarged at the start of doping but reduced with an increased dosage, which suggests a small amount of Co atoms tends to occupy the off-center of the SOD. These results can be understood as a coupling between spin impurities coupled with spinons and the chargons which gives rise to a charge redistribution and a reduced gap, a quantum spin liquid behavior in monolayer 1T MX_2 (M = Ta, Nb; X = Se, S). Instead, nonmagnetic impurities like K atoms shift the LHB bands away from the Fermi level and enlarge the energy gap for both monolayer $NbSe_2$ and $TaSe_2$ (Supplementary Fig. 6)[5].

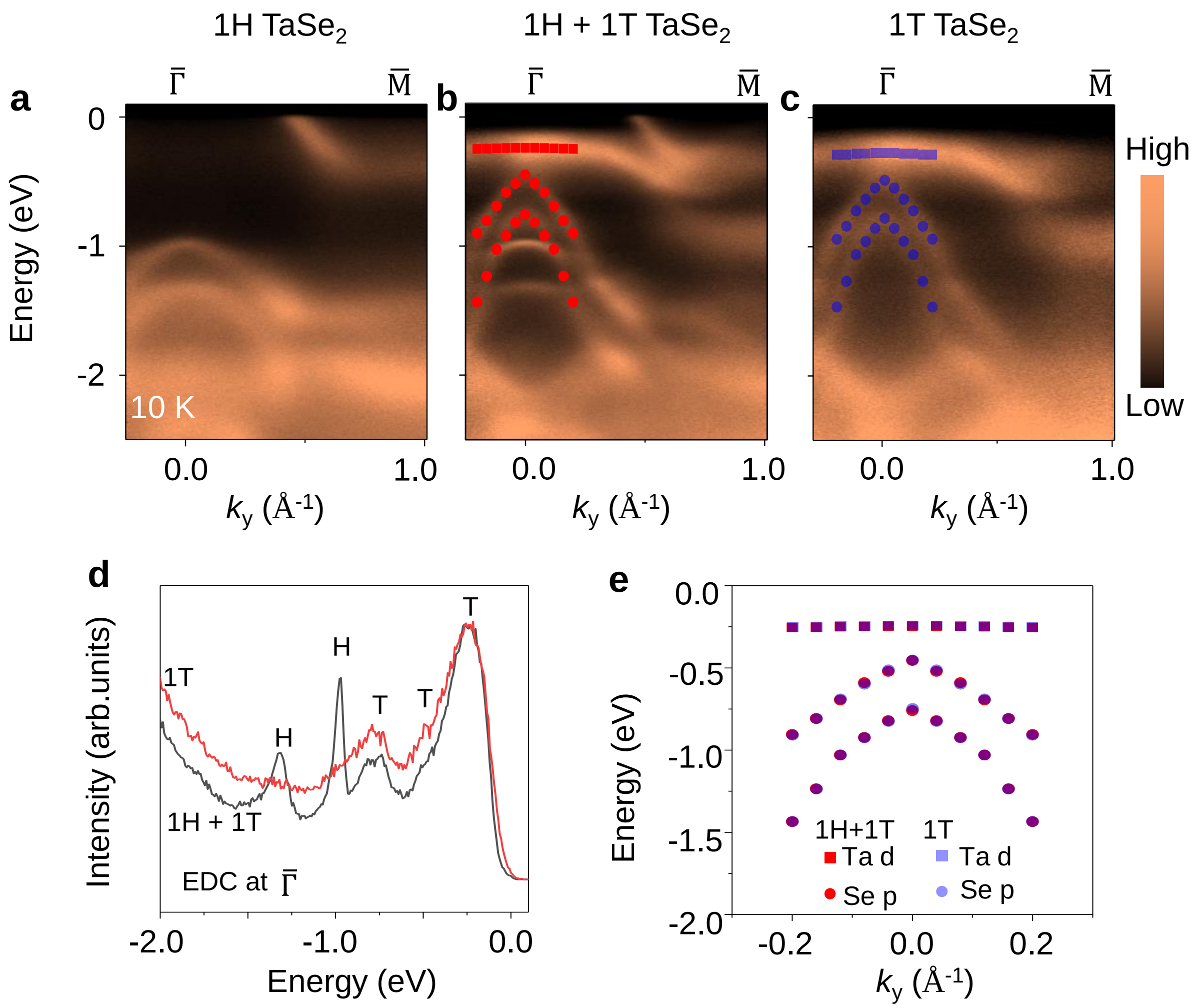

Supplementary Fig. 1 | ARPES spectra of monolayer $TaSe_2$ with varied growth temperatures. (**a-c**) ARPES spectra taken along the $\overline{\Gamma M}$ direction with He Iα ($h\nu$ = 21.2 eV) at 10 K on monolayer $TaSe_2$ grown at 500 °C (**a**), 650 °C (**b**), and 700 °C (**c**) show bands of 1H, 1H + 1T, and 1T phase, respectively. (**d**) Extracted EDCs for 1H + 1T mixed phase and 1T phase at the $\bar{\Gamma}$ point. The peaks from H phase and T phase are labeled. (**e**) The extracted band dispersions are plotted together for a comparison, showing the 1T phase bands are basically the same in the mixed phase and the pure phase.

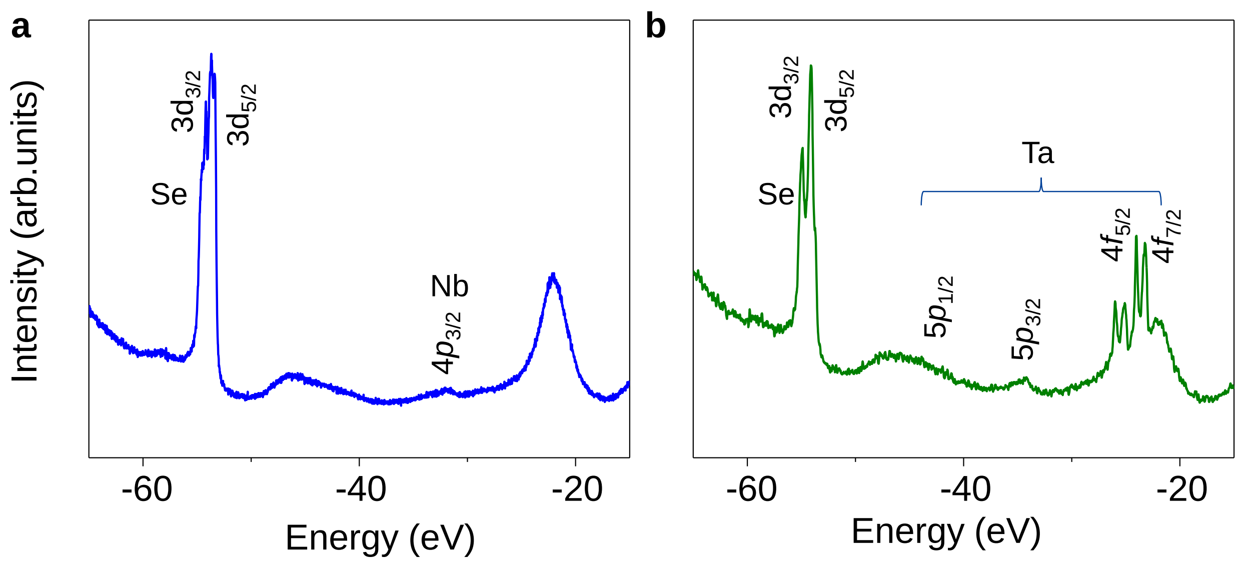

Supplementary Fig. 2 | Core-level scans on monolayer $NbSe_2$ and $TaSe_2$ films. (**a,b**) Photoemission spectra taken with 90 eV photons for a monolayer $NbSe_2$ (**a**), $TaSe_2$ film (**b**), respectively, show characteristic peaks of Nb, Ta, and Se core levels.

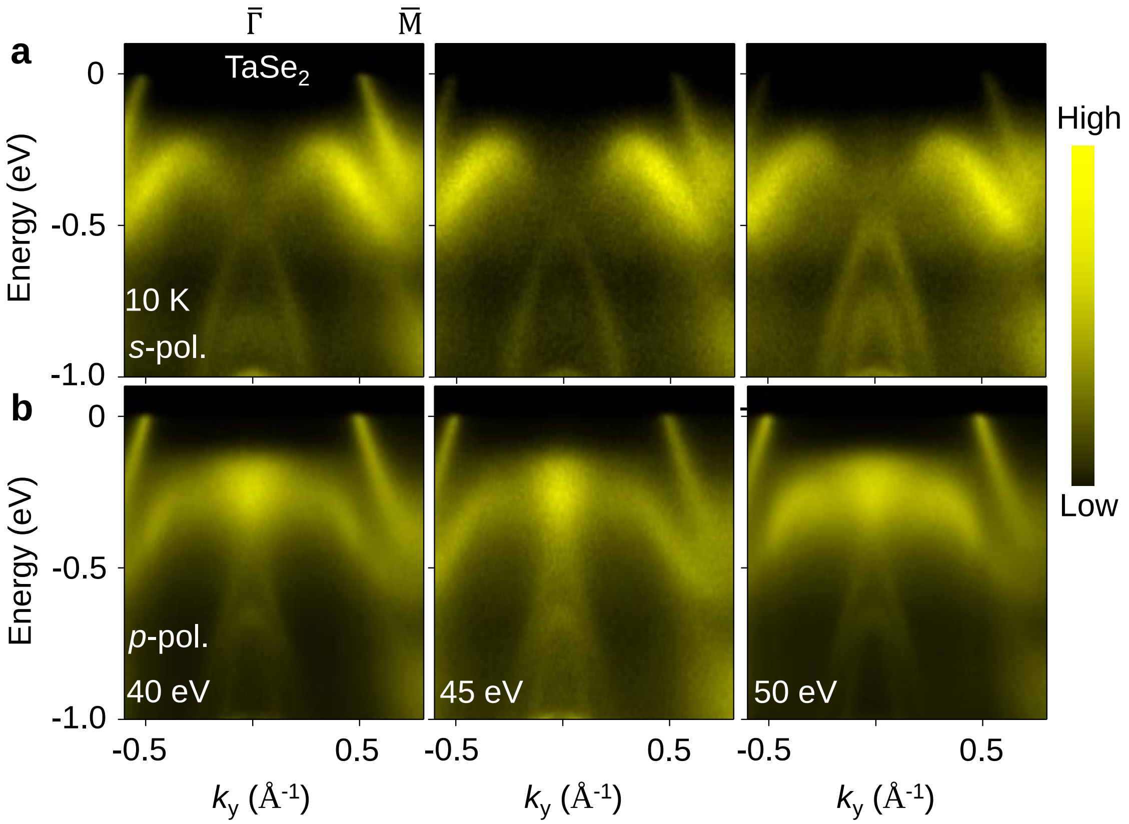

Supplementary Fig. 3 | Photon energy and polarization dependence of the ARPES spectra for monolayer $TaSe_2$. (**a,b**) ARPES maps taken at different photon energies along the $\overline{\Gamma M}$ direction at 10 K with *s* (**a**) and *p* (**b**) polarized light. The matrix element effects are evidenced in the spectra that LHB is more prominent in the *p* polarization measurements .

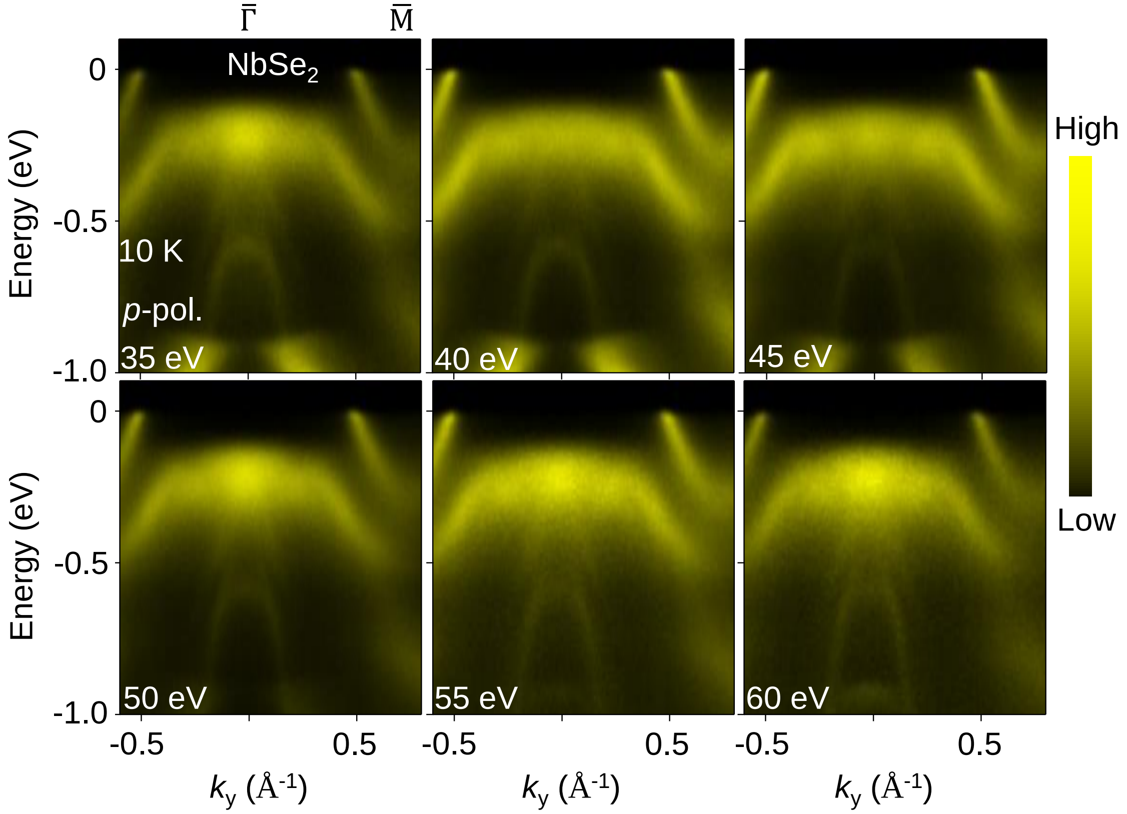

Supplementary Fig. 4 | Photon energy dependence of the ARPES spectra for monolayer $NbSe_2$. ARPES maps taken at different photon energies along the $\overline{\Gamma M}$ direction at 10 K with *p* polarized light. The ARPES spectra show no obvious photon energy dependence.

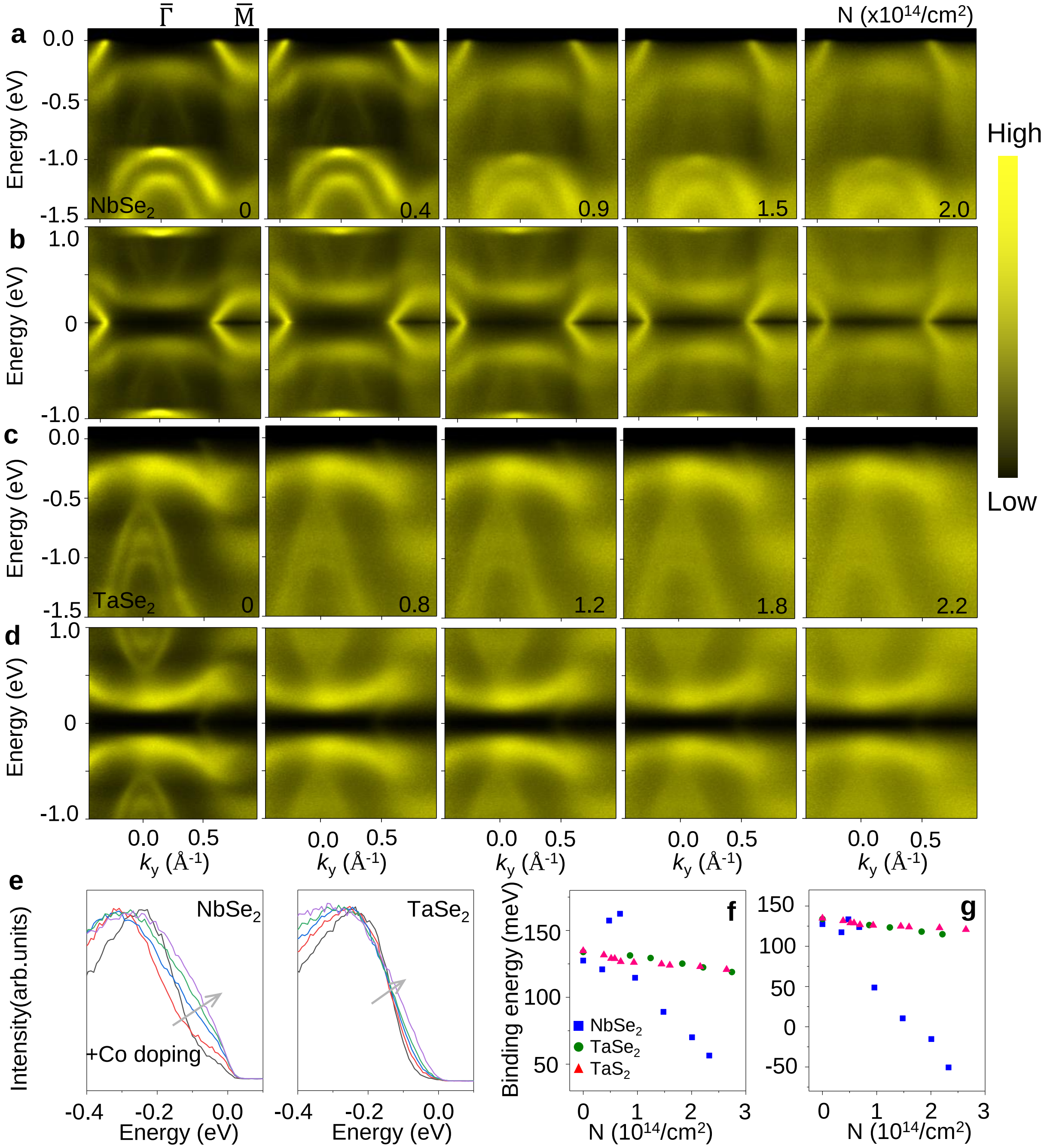

Supplementary Fig. 5 | Effect of surface Co doping on the band structure of monolayer $NbSe_2$ and $TaSe_2$. (**a,c**) Measured ARPES spectra taken along the $\overline{\Gamma M}$ direction at 10 K with Co doping on the surface of monolayer $NbSe_2$ (**a**) and $TaSe_2$ (**c**), respectively. Surface electron density (N)

determined from the Luttinger area of the Fermi surface is shown in each panel with a unit of 10^{14} cm^{-2}. (**b**,**d**) Corresponding symmetrized map to show the evolution of the flat band around the zone center with doping. (**e**) Normalized EDCs at the zone center with different doping levels. **(f)** The extracted doping dependence of the flat bands around the zone center determined by the leading-edge midpoints for monolayer $NbSe_2$, $TaSe_2$, and TaS_2, showing the flat band moves closer to the Fermi level with adding magnetic impurities. (**g**) The binding energy of the flat band as a function of surface carrier density with a correction of chemical potential shift determined from the shift of chalcogenide states with doping.

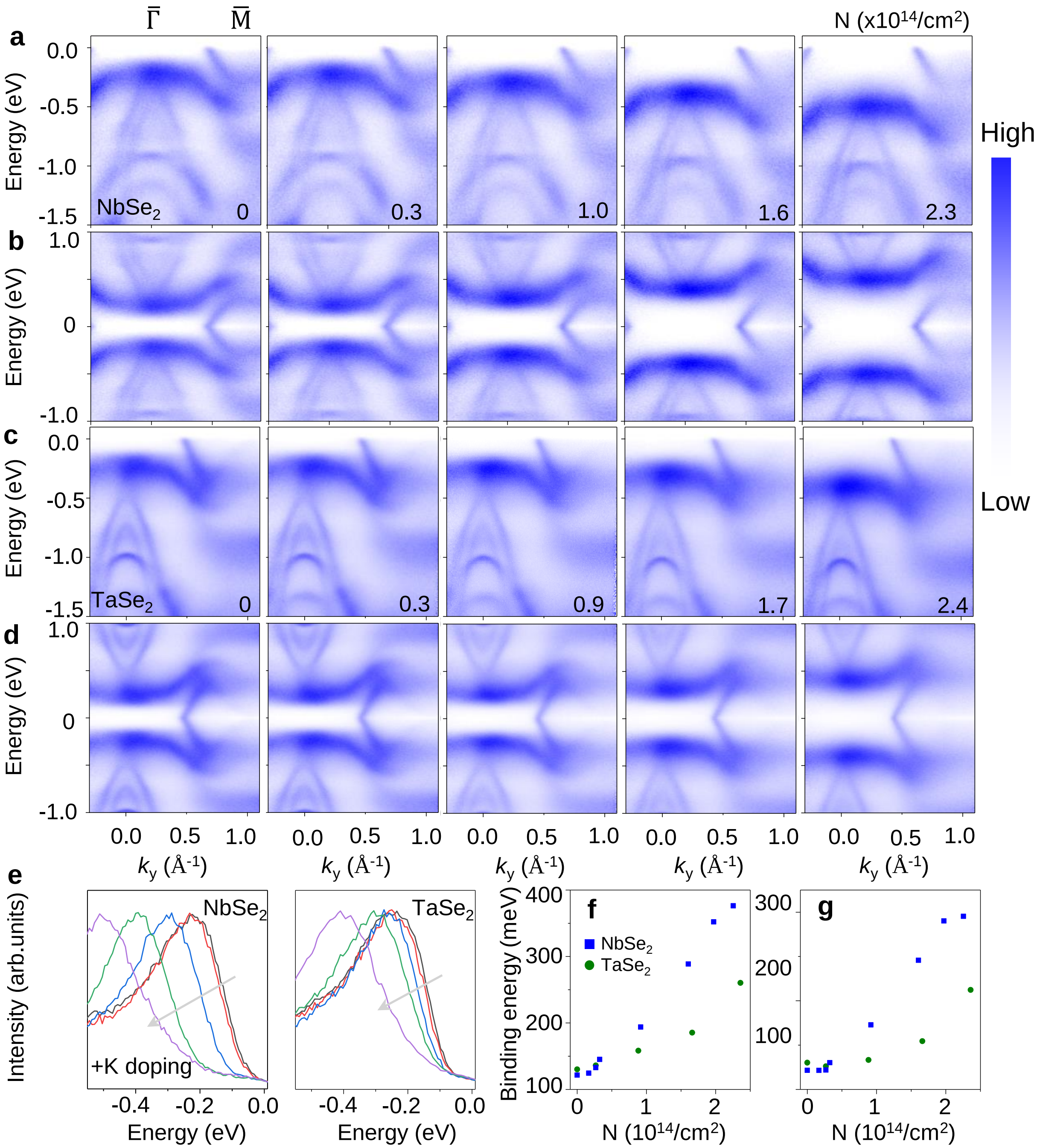

Supplementary Fig. 6 | Effect of surface K doping on the band structure of monolayer $NbSe_2$ and $TaSe_2$. (**a**,**c**) Measured ARPES spectra taken along the $\overline{\Gamma M}$ direction at 10 K with K doping on the surface of monolayer $NbSe_2$ (**a**) and $TaSe_2$ (**c**), respectively. Surface electron density (N) determined from the Luttinger area of the Fermi surface is shown in each panel with a unit of 10^{14}

cm^{-2}. (**b**,**d**) Corresponding symmetrized map to show the evolution of the flat band around the zone center with doping. (**e**) Normalized EDCs at the zone center with different doping levels. (**f**) The extracted doping dependence of the flat bands around the zone center determined by the leading-edge midpoints for monolayer $NbSe_2$, $TaSe_2$, and TaS_2. (**g**) The binding energy of the flat band as a function of surface carrier density with a correction of chemical potential shift determined from the shift of chalcogenide states with doping.

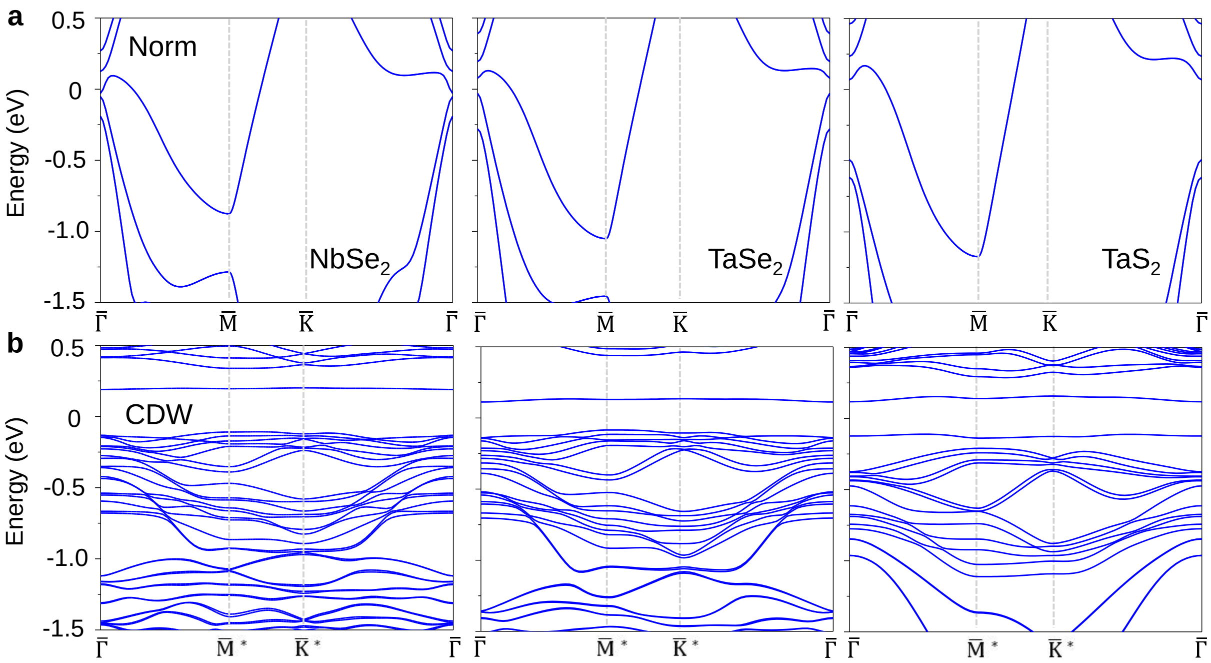

Supplementary Fig. 7 | DFT band dispersions for the (1 × 1) and ($\sqrt{13} \times \sqrt{13}$) structures of monolayer $NbSe_2$, $TaSe_2$, and TaS_2. (**a,b**) Calculated band structures for (1 × 1) structure (**a)** in the normal state and ($\sqrt{13} \times \sqrt{13}$) superstructure (**b**) in the Mott-CDW state of these three compounds with Hubbard U included.

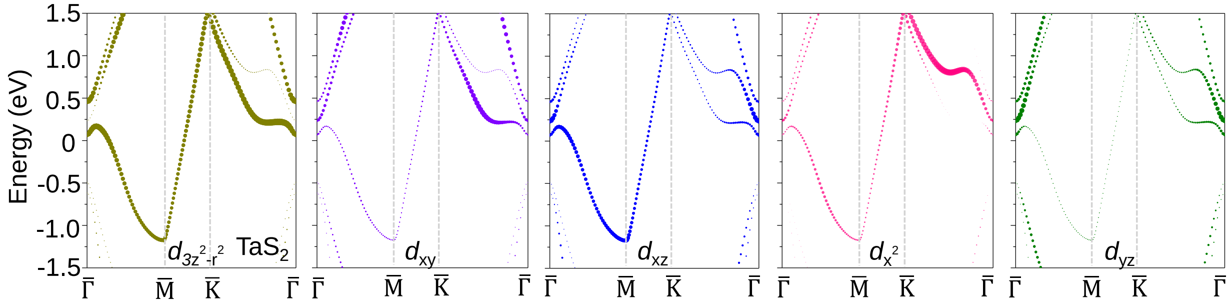

Supplementary Fig. 8 | Projected spectral weight for the (1 × 1) structure of monolayer TaS_2. Calculated spectral weight projected on the five *d* orbitals from the central Ta atom of monolayer TaS_2 for the (1 × 1) structure in the normal state.

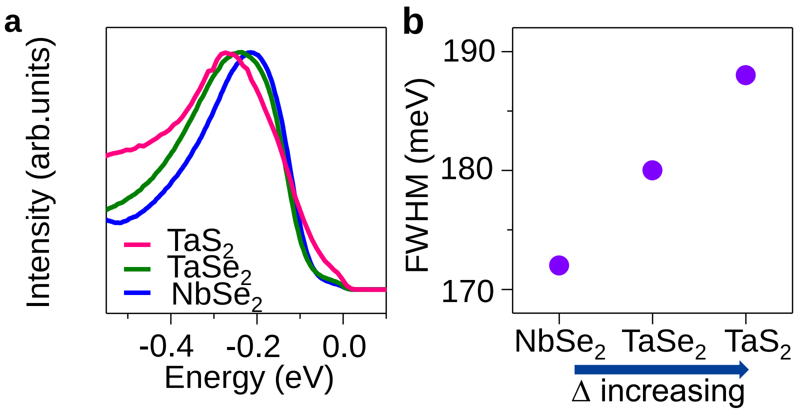

Supplementary Fig. 9 | Integrated EDCs and FWHM. (**a**) Integrated EDCs for monolayer 1T-TaS_2, 1T-$NaSe_2$, and 1T-$NbSe_2$ around the $\bar{\Gamma}$ point (±0.2 Å). (**b**) Summary of the extracted FWHM of the integrated EDCs from the three systems, plot against the energy gap. Different photon energies are checked to reduce impact of the matrix element effect in ARPES spectra. Error bars of the FWHM are estimated from the uncertainty in the fitting of the EDCs.

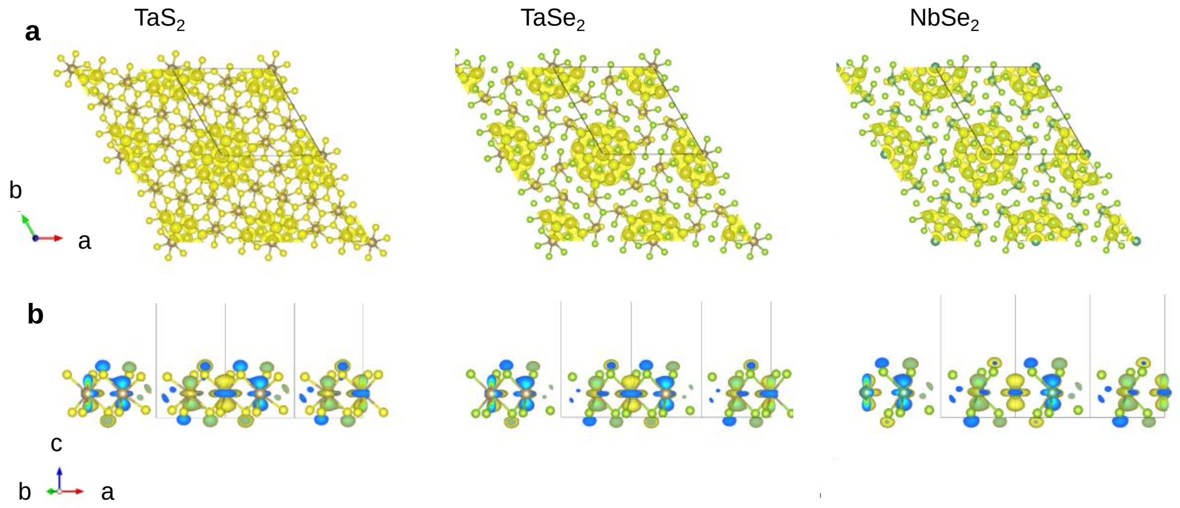

Supplementary Fig. 10 | Illustration of electron density in real-space for the LHB at the $\bar{\Gamma}$ point. (**a,b**) Orbital textures in the *ab*-plane (**a**) and *ac*-plane (**b**) for the $(\sqrt{13} \times \sqrt{13})$ structure in the CDW state of monolayer TaS_2, $TaSe_2$, and $NbSe_2$.

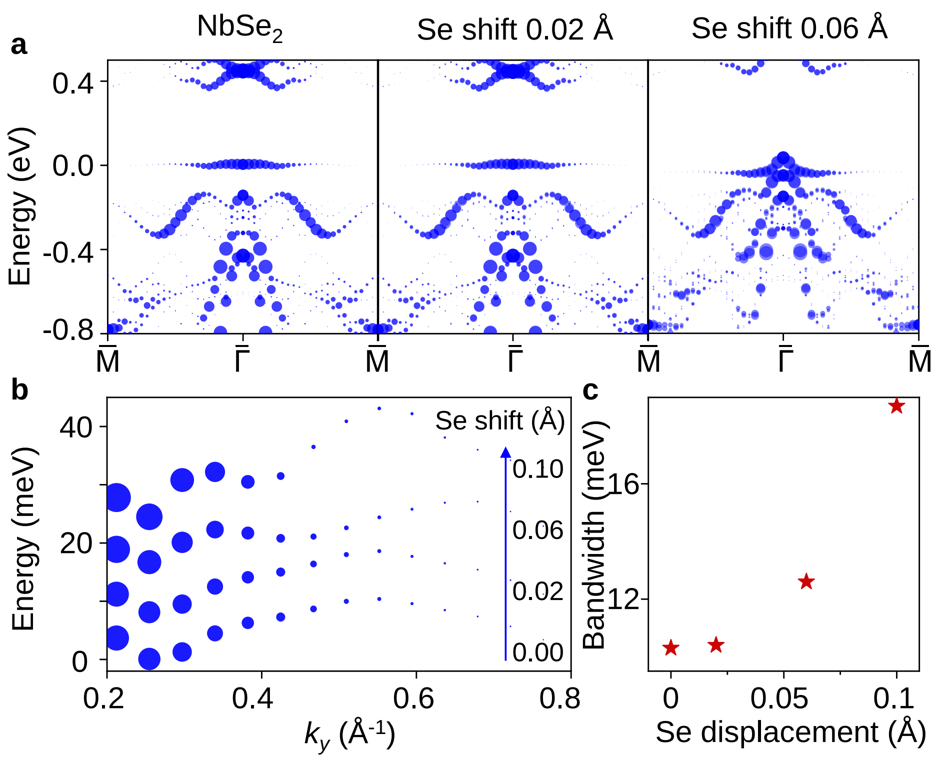

Supplementary Fig. 11 | Calculated band structure with different Se displacements. (**a**) Calculated unfolded band dispersions for monolayer 1T-$NbSe_2$ with the ($\sqrt{13}\times\sqrt{13}$) nonmagnetic superstructure by imposing various Se atomic displacements while keeping the lattice constant of the unit cell unchanged. Se atoms were shifted along z axis to reserve the lattice symmetry. (**b**) Zoom-in view of the dispersions for the projected $d_{3z^2-r^2}$ orbitals of the central Nb atom. As the Se bands penetrate the Nb *d* bands around the $\bar{\Gamma}$ point at larger displacements and affect the determination of the bandwidth, we obtained the bandwidth of the metal band starting from 0.2 Å^{-1}. (**c**) The extracted bandwidth of the metal band, plotted against the Se atomic displacements, showing the increased bandwidth of the Hubbard *d* band with increasing Se displacements.

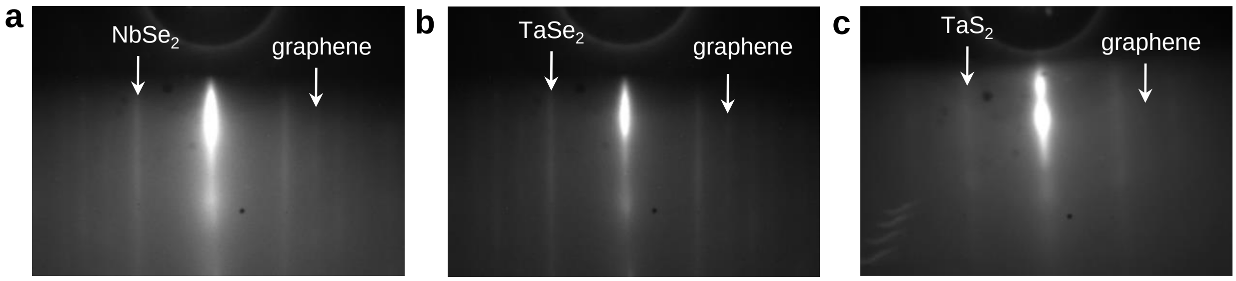

Supplementary Fig. 12 | RHEED patterns. (**a-c**), RHEED patterns taken at room temperature for monolayer (**a**) $NbSe_2$, (**b**) $TaSe_2$, and (**c**) TaS_2, respectively.

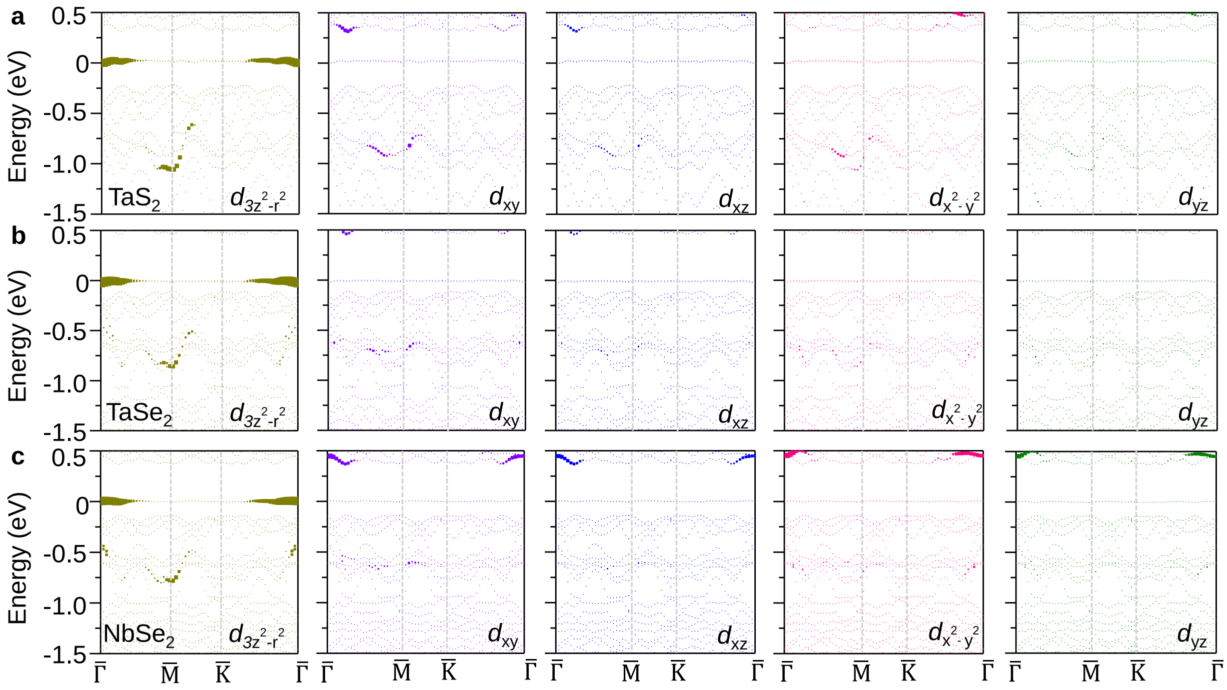

Supplementary Fig. 13 | Calculated unfolded band dispersions for the ($\sqrt{13}\times\sqrt{13}$) superstructure of monolayer $NbSe_2$, $TaSe_2$, and TaS_2 films. (**a-c**) Calculated spectral weight projected on the five *d* orbitals from the metal atoms for the ($\sqrt{13}\times\sqrt{13}$) superstructure in the CDW phase. The spectral weight of $d_{3z^2-r^2}$ is ~90%-95% of the total spectral weight of the Hubbard band in the three compounds.

Supplementary References

1. Law, K. T. & Lee, P. A. 1T-TaS_2 as a quantum spin liquid. *Proc. Natl. Acad. Sci.* **114**, 6996-7000 (2017).

2. Balents, L. Spin liquids in frustrated magnets. *Nature* **464**, 199-208 (2010).

3. He, W. Y. et al. Spinon fermi surface in a cluster Mott insulator model on a triangular lattice and possible application to 1T-TaS_2. *Phys. Rev. Lett.* **121**, 046401 (2018).

4. He, W. Y. & Lee, P. A. Electronic density of states of a U(1) quantum spin liquid with spinon Fermi surface. I. Orbital magnetic field effects. *Phys. Rev. B* **107**, 195155 (2023).

5. Chen, H. et al. Spectroscopic Evidence for Possible Quantum Spin Liquid Behavior in a Two-Dimensional Mott Insulator. *Phys. Rev. Lett*. **134**, 066402 (2025).
